# Polyhydroxybutyrate-producing cyanobacteria from lampenflora: The case study of the “Stiffe” caves in Italy

**DOI:** 10.3389/fmicb.2022.933398

**Published:** 2022-07-28

**Authors:** Rihab Djebaili, Amedeo Mignini, Ilaria Vaccarelli, Marika Pellegrini, Daniela M. Spera, Maddalena Del Gallo, Anna Maria D’Alessandro

**Affiliations:** ^1^Department of Life, Health and Environmental Sciences, University of L’Aquila, L’Aquila, Italy; ^2^Quality Engineering S.r.l., Pescara, Italy

**Keywords:** microbial communities of caves, deep biosphere, biopolymers, polyhydroxyalkanoates, 16S rRNA gene metabarcoding

## Abstract

This study aimed to estimate the green formation lampenflora of “Stiffe” caves in order to evaluate their suitability as an isolation source of cyanobacteria useful for the production of polyhydroxyalkanoates (PHAs). The cave system was chosen as the sampling site due to its touristic use and the presence of high-impact illuminations. The biofilms and the mats of the illuminated walls were sampled. Samples were investigated by 16S rRNA gene analysis and culturable cyanobacteria isolation. The isolated strains were then screened for the production of PHAs under typical culturing and nutritional starvation. Cultures were checked for PHA accumulation, poly-β-hydroxybutyrate (PHB) presence (infrared spectroscopy), and pigment production. The 16S rRNA gene metabarcoding. Highlighted a considerable extent of the pressure exerted by anthropogenic activities. However, the isolation yielded eleven cyanobacteria isolates with good PHA (mainly PHB)-producing abilities and interesting pigment production rates (chlorophyll a and carotenoids). Under normal conditions (BG11_0_), the accumulation abilities ranged from 266 to 1,152 ng mg dry biomass^–1^. The optimization of bioprocesses through nutritional starvation resulted in a 2.5-fold increase. Fourier transform infrared (FTIR) studies established the occurrence of PHB within PHAs extracted by cyanobacteria isolates. The comparison of results with standard strains underlined good production rates. For C2 and C8 strains, PHA accumulation rates under starvation were higher than *Azospirillum brasilense* and similar to *Synechocystis* cf. *salina* 192. This study broadened the knowledge of the microbial communities of mats and biofilms on the lightened walls of the caves. These findings suggested that these structures, which are common in tourist caves, could be used to isolate valuable strains before remediation measures are adopted.

## Introduction

Due to the involvement of the underground environments in various biological processes and the great scenarios present within them, many caves across the world have been converted into scientific laboratories and tourist attractions. These caves undergo several environmental transformations due to paths’ construction, visitors’ presence, and artificial lighting installation. These changes modify the caves’ physicochemical conditions (Cigna, 2019; Piano et al., 2021), with significant alterations to biotic and abiotic components (Lim et al., 2018; Šebela et al., 2019; Nicolosi et al., 2021; Piano et al., 2021). Visitors’ presence changes the local microclimate and introduces fungal spores and bacteria into the cave environment (Mammola et al., 2017; Novas et al., 2017; Zhelyazkova et al., 2020). Humidity, temperature, CO_2_ levels, and electrical lighting enhance the growth of specific photosynthetic communities known as lampenflora in cave entrances and the speleothem (Piano et al., 2015; Nikolić et al., 2020). Generally, microbial communities in cave entrances constitute biofilms (Albertano, 2012), where there is also the presence of cyanobacteria and microalgae (Poulíčková and Hasler, 2007; Czerwik-Marcinkowska, 2013; Lamprinou et al., 2014). Lampenflora typically causes biodeterioration of colonized surfaces (Figueroa et al., 2017; Muñoz-Fernández et al., 2021), and a variety of approaches (i.e., physical, mechanical, and chemical) are utilized to limit photosynthesis and propagation (Mulec and Kosi, 2009; Cigna, 2016; Figueroa et al., 2017). These strategies to devise the existence of lampenflora have been studied for many years. Few studies, however, have investigated the traits that microbes develop in these harsh conditions and whether they can act as a source of interesting molecules.

In this study, we have focused our attention on the cyanobacteria of lampenflora. Cyanobacteria are an ancient lineage of slow-growing ubiquitous photosynthetic prokaryotes found in a wide range of terrestrial and aquatic lightened environments (Abed and Garcia-Pichel, 2001; Abed et al., 2009; Lemes-da-Silva et al., 2011; Panou and Gkelis, 2022). Cyanobacteria are the most important primary producers on Earth, including extreme environments (Gan and Bryant, 2015). They are well adapted to extreme environments due to their ability to withstand high osmotic pressure, low temperatures, arid conditions, and UV radiations (Sechrest and Brooks, 2002; Satyanarayana et al., 2005; Chrismas et al., 2015; Rasouli-Dogaheh et al., 2022). Many authors have described their presence in caves as most close to entrances lit by direct or indirect sunlight or by artificial light in those open to tourists (Pentecost, 1992; Giordano et al., 2000; Asencio and Aboal, 2011; Czerwik-Marcinkowska and Mroziñska, 2011; Albertano, 2012; Czerwik-Marcinkowska, 2013; Pfendler et al., 2018; Puente-Sánchez et al., 2018; Behrendt et al., 2020; Havlena et al., 2021; Panou and Gkelis, 2022).

Numerous new bioactive compounds and polymers have been identified in cyanobacteria belonging to different environments (Abarzua et al., 1999; Shimizu, 2003; Dahms et al., 2006) produced, in particular, in response to environmental changes and biotic and abiotic stresses, providing protection and promoting survival (Singh and Mallick, 2017). Among polymers, cyanobacteria produce polyhydroxyalkanoates (PHAs) (Balaji et al., 2013; Koch et al., 2020). PHAs are lipoid materials accumulated by a wide variety of microorganisms in the presence of abundant carbon sources, which can have various uses, including the production of bioplastics (Anderson and Dawes, 1990; Abed et al., 2009). The most common PHA in prokaryotic cells is poly-β-hydroxybutyrate (PHB), an abundant energy and carbon source storage material (Balaji et al., 2013). Several heterotrophic bacteria, such as *Cupriavidus necator* and *Escherichia coli*, can produce PHB by fermentation (Liebergesell et al., 1994; Kichise et al., 1999; Abed et al., 2009; Wang et al., 2013; Ansari and Fatma, 2016; Utharn et al., 2021). However, these production processes use organic carbon sources mainly derived from crops (Koch et al., 2020). Cyanobacteria are a promising alternative for PHB production (Balaji et al., 2013). Cyanobacteria have minimal nutrient requirements for growth and accumulate PHAs through oxygenated photosynthesis (Singh and Mallick, 2017). Under nutrient-limited conditions, such as nitrogen starvation, cells enter a quiescent state known as chlorosis (Koch et al., 2020). Cyanobacteria degrade their photosynthetic apparatus during chlorosis. Beyond this degradation, the accumulation of large amounts of glycogen for carbon and energy storage occurs. At the end of the process, the cells begin to degrade glycogen and convert it into PHB (Koch et al., 2019).

Given the anthropogenic pressure present in the “Stiffe” touristic cave (L’Aquila, Italy), we hypothesized that cyanobacterial strains within artificial lightened walls’ biofilms and mats might serve as good producers of PHB. To examine the bacterial and archaeal communities’ composition of green formations of lighted walls, we investigated a global sample with 16S rRNA gene metabarcoding. To investigate the suitability of these formations as an isolation source of industrially valuable microbial strains, we carried out samplings from five sites inside this cave, and we performed an isolation of cyanobacteria. Strains were studied for the PHA and pigment production abilities under normal and nutritional starvation conditions. PHAs recovered were further characterized by infrared spectroscopy.

## Materials and methods

### “Stiffe” caves site

The sampling was carried out in the Stiffe caves (42°15′20.62″ North; 13°32′32.51″ East; 695 m Altitude). It consists of complex hydrologically active karst paths, with a vertical cave development of + 186 m from the entrance. The Stiffe caves are about 130 km from Rome. At present, the Stiffe caves receive around 45,000 tourists a year and have been open to the public since the 1990s. The accessible touristic pathway includes artificial tunnels and footbridges. Currently, the total length of the show path represents 1 km from the total known layout, which is approximately 2.3 km. The external light covers the first 20 m and gradually gives way to artificial lighting systems that have been installed both along the tourist way and around inaccessible tourist areas illuminating different morphologies and concretions. In the beginning of the 1990s, the lighting system of the Stiffe caves was designed following a technical study counting the environmental impact to prevent the green formations near the light sources. Illuminated areas were not directly visible on the tourist path, with the initially lighting color consisting of a warm and bright light like the natural one. The underground stream was lit directly with warmer light. According to earlier technical reports, wood’s lights illuminated some concretions. Subsequently, the mismanagement of lighting systems led to the proliferation of many photosynthetic communities on illuminated limestone walls, including vascular plants throughout the tourist way. The lamps inside the cave are of different types and colors, and so far, no steps have been taken to set low-impact lighting.

### Collection of the samples

**FIGURE 1:**
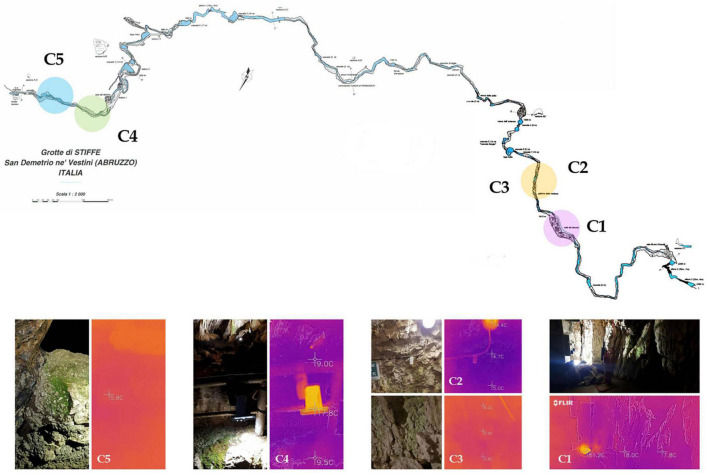
Cave survey of the “Stiffe” caves. Sampling areas are highlighted in the plan view and pictures on the bottom show the thermic camera and normal photos for each site.

The sampling was performed in May 2021 in a period of cave closure. According to the measures adopted to contain the COVID-19 spread, touristic visits were interrupted from March to July 2020 and from November to June 2021. Except for ordinary and extraordinary maintenance work, the whole internal lighting stayed off during the cave closures. Before sampling, portable probes were used to record temperature, relative humidity (TACKLIFE HM03), and light intensity (URCERI portable lux meter). A handheld thermal camera (FLIR One Android USB-C) was used to acquire the thermic photos of each site, measuring the temperature lamp and the temperature variations. Samples from photosynthetic biofilms were then collected from five different sites near lighting systems along the tourist walkway. They were swabbed from the surface with a sterile knife, collected in sterility, and stored in refrigerated containers. The samples for the isolation of cyanobacteria were processed as soon as they were brought to the laboratory. For the DNA isolation, three samples from each site were collected and then directly pooled together in the same tube (Sample C6). According to the manufacturer’s instructions, this global sample was stabilized with a solution of RNAlater (Ambion, Austin, TX, United States) and stored at −80°C until processed. Sample site characteristics are shown in Table 1, while sampling site localization and photos are depicted in Figure 1. On average, in sampling sites, there was a temperature of 13.7°C and a relative humidity of 73.8%. All samples grew on limestone walls subjected to any water scrolling or dripping. On average, the light intensity of the light source was 186.15 lux, while the samples received a 13.65 lux exposure (except for sample C5).

**TABLE 1:** Cyanobacterial isolates and environmental variables measured at the different sampling sites of “Stiffe” caves.

Strain ID	Sampling site	Site temperature (°C)	RH (%)	LS (Lux)	
				Light system	Sample
C1	C1 – A parietal biofilm adheres to the concrete in the innermost part of the cave.	15.0	67.6	280.0	10.0
C2					
C3	C2 – On the concrete along the walls of the last man-made tunnel.	15.1	67.6	220.0	20.0
C4					
C5	C3 – A parietal biofilm.	13.0	75.1	120.0	10.0
C6					
C7	C4 – Mosses from the photosynthetic formation.	12.6	80.2	124.6	14.6
C8					
C9					
C10	C5 – No functioning light site.	12.9	78.5	−	−
C11					

### 16S rRNA gene metabarcoding

To assess the overall lampenflora bacterial and archaeal communities, three replicates were taken from the C6 global sample and subjected to DNA extraction. Briefly, 500 mg was processed by NucleoSpin^®^Soil kit (Macherey-Nagel, Germany). To determine DNA content and purity, extracted samples were subjected to spectrophotometric and fluorometric examination utilizing a NanoDrop spectrophotometer (Thermo Scientific™) and a Qubit fluorometer (Thermo Scientific™). Using paired-end 16S rRNA gene community sequencing on the MiSeq Illumina platform, a specific 16S rRNA gene technique was performed to amplify bacteria and archaea (Bio-Fab Research, Italy). Using the analytical approach previously reported (Vaccarelli et al., 2021), we focused on the V3 and V4 regions of 16S rRNA gene (Mizrahi-Man et al., 2013; Choi et al., 2020). After filtering, the reads were examined for quality and counted. The DADA2 plugin in QIIME2 (qiime2-2020.2 version) was utilized for amplicon sequence variant (ASV) assembly (Bolyen et al., 2019). The V3-V4 specific region was extracted from the 16S file retrieved from the SILVA 132 database^1^ and utilized for classifier training by the fit-classifier-naive-Bayes plugin. For the taxonomic assignment, a 97% similitude was used.

### Cyanobacteria isolation

For cyanobacterial isolation, several dilutions up to 10^–4^ were prepared from each sample and plated on BG11 (H_3_BO_3_ 0.003, CaCl_2_ 2H_2_O 0.036, C_6_H_8_O_7_ 0.006, Co (NO_3_)_2_ 6H_2_O 0.00005, CuSO_4_ 5H_2_O 0.00008, EDTA 0.001, C_6_H_8_FeNO_7_ 0.006, MgSO4 7H_2_O 0.075, MnCl_2_ 4H2O 0.002, K_2_HPO_4_ 3H_2_O 0.04, Na_2_CO_3_ H_2_O 0.04, Na_2_MoO_4_ 2H_2_O 0.0004, NaNO_3_ 1.5, ZnSO_4_ 7H_2_O 0.0002, H_2_O 1,000 ml, and pH 8.5 ± 2) (Allen and Stanier, 1968). Enrichment cultures for all the samples were also prepared in liquid BG11 (1:10 ratio). Liquid and solid cultures were incubated for 1 week at 28°C and a 12-h photoperiod lightening of 150–200 μmol (photon) m^–2^ s^–1^. After incubation, enrichment cultures were plated on solid BG11 following the same procedure. Biomass clumps developed on plates were subcultured several times until individual colonies appeared pure. Putative cyanobacteria colonies were examined with optical microscopy (LEICA DME) to provide preliminary identification based on the morphotype. Each compliant isolate received an identification code (ID). In total, we obtained eleven isolates (C1–C11). The isolated strains were cultured on a liquid BG11 medium in flasks (500 ml) and small bioreactors (a 500-ml glass bottle equipped with GLS 45 screw cap with three-port lids and filled with 250 ml of BG11). After uniformity and purity checks, isolates were stored at the Environmental Microbiology culture collection (LMUNIVAQ) in BG11 agar slants and glycerolates (50% v/v, -80°C storage).

### Polyhydroxyalkanoate production

The different cyanobacterial strains were cultured on a BG11_0_ liquid medium for 5 days under optimal growth conditions (28°C, 120 rpm, and constant illumination of 40–50 μmol of photons m^–2^s^–1^). Exponentially growing cells (OD 0.4–0.8) were harvested by centrifugation (4,000 *g* for 10 min). The starvation was induced by suspending the pellet in a modified BG11 medium (devoid of basic nutrients) until reaching an OD of 0.4 (for nitrogen starvation, BG11 without NaNO_3_; for phosphorus deprivation, BG11 supplemented with KCl instead of K_2_HPO_4_; and for sulfur deprivation, BG11 supplemented with MgCl instead of MgSO_4_) and incubated under the same conditions until the late log phase of growth for approximately 20 days. After incubation, the calcium acetate (10 mM) was added to the cultures as an organic carbon source and incubated under the same growth conditions for another 10 days. Cultures are then extracted and quantitated for PHB production (Koch et al., 2019, 2020; Utharn et al., 2021). To compare the PHA production rates recorded for isolates, we quantified the PHAs produced by *Synechocystis* cf. *salina* 192 (CCALA 192), *Azospirillum brasilense* Cd (ATCC 29729), and *Halomonas eurihalina* (DMSZ 5710), bacteria known for their PHA-producing abilities.

### Polyhydroxyalkanoate extraction and quantification

The cells’ dry pellets, obtained by centrifugation of 10 ml from each liquid culture, were screened for the production of PHAs. The biomass was placed in a thermostatic bath at 100°C for 1 min; after cooling, the samples were placed in the freezer (−20°C) for 2 h and dried at 90°C until constant weight. According to the method of Zilliges and Damrow (2017), PHB extraction was realized by the PHB hydrolysis into its monomer (R)-3-hydroxybutyric acid (R-3-HB). Briefly, 300 μl of NaOH (0.5 N) was introduced into glass tubes containing 5 mg of each dry sample and incubated in an ultrasonic bath at 85°C for 1 h. After a rapid cooling on ice, the samples were neutralized with the addition of 100 μl of HCl (1 N) and vortexed for a few seconds. Later, centrifugation was performed for 1 min/4,500 *g* to remove the cell debris, and the supernatant of each sample was transferred to 1.5 ml tubes. The PHA concentration was determined by an enzymatic test using the Beta-Hydroxybutyrate Assay Kit (Sigma Aldrich).

### Polyhydroxyalkanoate characterization by attenuated total reflectance-fourier transform infrared

The PHA extracted functional groups were characterized by attenuated total reflectance-Fourier transform infrared (ATR-FTIR) spectroscopy (Bruker Vertex 70 V), using a spectral range of 4,000–400 cm^–1^. The powders of the PHA extracted samples were placed against the ATR crystal, the system was vacuumed (up to ∼ 2 hPA), and the spectra were scanned using a resolution of 4 cm^–1^ and 64 scans. Acquired spectra were processed and studied using the SpectraGryph version 1.2.15 software. The spectra of PHAs extracted from “Stiffe” isolates were merged. The spectrum obtained was compared to the *Synechocystis* cf. and the PHB standard ones (Sigma-Aldrich, St. Louis, MI, United States).

### Determination of photosynthetic pigment contents

Chlorophyll a and carotenoid contents in each strain were determined according to the protocol published by Zavrel and collaborators (Zavřel et al., 2015). Briefly, a volume of 1 ml of cyanobacterial culture suspension in the stationary phase was centrifuged at 15,000 *g* for 7 min to recover the pellet. Then, 1 ml of methanol previously cooled at + 4°C was introduced into each tube. The samples are then vortexed, covered with aluminum foil, and incubated at + 4°C for 20 min to extract the cells’ pigments. Centrifugation at 15,000 *g* for 7 min was performed (visually checking the bluish/purple coloration of the pellet). Then, the absorbance of each sample was measured at 470, 665, and 720 nm.

### Statistical analysis

All experimental data are the mean of three replicates ± standard deviation. Statistical significance between groups was evaluated by one-way analysis of variance (ANOVA) followed by Fisher’s LSD *post hoc* test, comparing mean values at a 5% level of significance (*p* < 0.05). Differences between the two groups were investigated by Student’s *t*-test. All statistical calculations were performed using the XLSTAT 2016 software (Addinsoft, Paris, France).

## Results

### 16S rRNA gene metabarcoding

**FIGURE 2:**
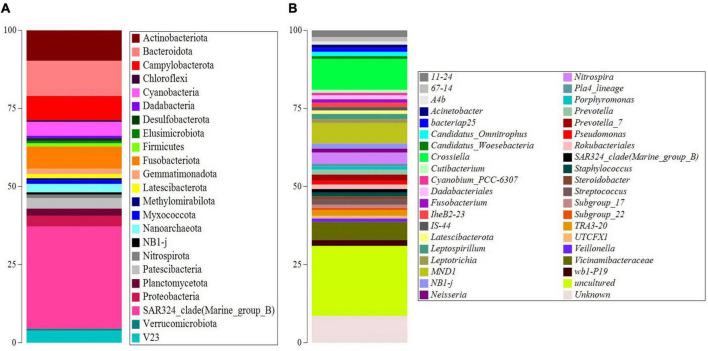
Taxonomy barplots of the main ASVs (abundances > 0.5%) at phylum **(A)** and genus level **(B)**.

The 16S rRNA gene metabarcoding generated a total number of 30,200 ASVs. Results were first used to calculate the alpha-diversity metrics. The indexes showed 1,721 taxa, with the same value as the calculated Chao-1 index (1,721). Both Simpson 1-D (0.9959) and Shannon H’ (6.657) indexes underlined high diversity. ASVs were then filtered to retain values over > 0.5% and studied at the different taxonomic levels. The sample was mainly constituted by bacteria at the domain level, accounting for 98.6% of the total abundances, while archaea accounted for the left 1.4%. Figure 2 and Supplementary Tables 1, 2 of Supplementary Material show the abundance of the main taxonomic levels. At phylum level (Figure 2A and Supplementary Table 1), the SAR324_clade (Marine_group_B) constituted 32.6%, followed by Bacteroidota (11.0%), Actinobacteriota (9.4%), Campylobacterota (7.5%), Fusobacteriota (6.9%), Cyanobacteria (4.3%), Patescibacteria (3.3%), Proteobacteria (3.3%), and Planctomycetota (2.3%). At genus level (Figure 2B and Supplementary Table 2), the ASVs were mainly composed of uncultured taxa (22.5%) mainly associated with Proteobacteria phylum (Gammaproteobacteria), followed by *Crossiella* (9.9%), and unknown taxa (8.6%) mainly associated with Proteobacteria phylum (Gammaproteobacteria, Enterobacterales, and Pasteurellaceae), *MND1* (6.5%), *Vicinamibacteracea* (5.9%), and *Nitrospira* (3.5%). Among the ASVs with abundances less than 2%, an important result obtained was the presence of *Cyanobium*_PCC-6307 (0.8%).

### Strains’ isolation and identification

Strains’ isolation and purification on BG11 agar medium were used to obtain eleven strains with different morphologies. Two strains were isolated from each site, except for sample site 4, which yielded three strains. The isolation source of each strain is reported in Table 1. Supplementary Figure 1 represents the photos obtained with the microscopic observations of the strains. Based on these observations, the strains were putatively associated with *Synechocystis* (strains C2, C3, C8, and C9) and *Synechococcus* (strains C1, C4, C5, C6, C7, C10, and C11).

### Polyhydroxyalkanoate production, optimization, and quantification

**FIGURE 3:**
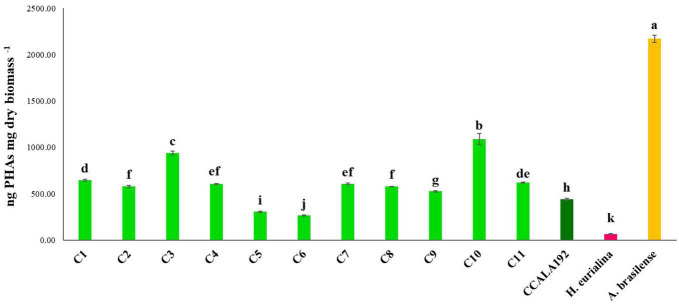
Polyhydroxyalkanoates contents recorded for “Stiffe” caves’ cyanobacteria and *Synechocystis* cf. *salina*, grown in BG11_0_, and for *A. brasilense* and *H. eurihalina*, cultured on NFCC and MH, respectively. Uppercase letters refer to the comparison among BG11_0_ growths and *A. brasilense* and *H. eurihalina.* Lowercase letters refer to the comparison among BG11 starving growths and *A. brasilense* and *H. eurihalina.* For both conditions, results followed by the same case letters are not significantly different according to Fisher’s LSD *post hoc* test (*p* > 0.05) (LSD value, 40.3).

**FIGURE 4:**
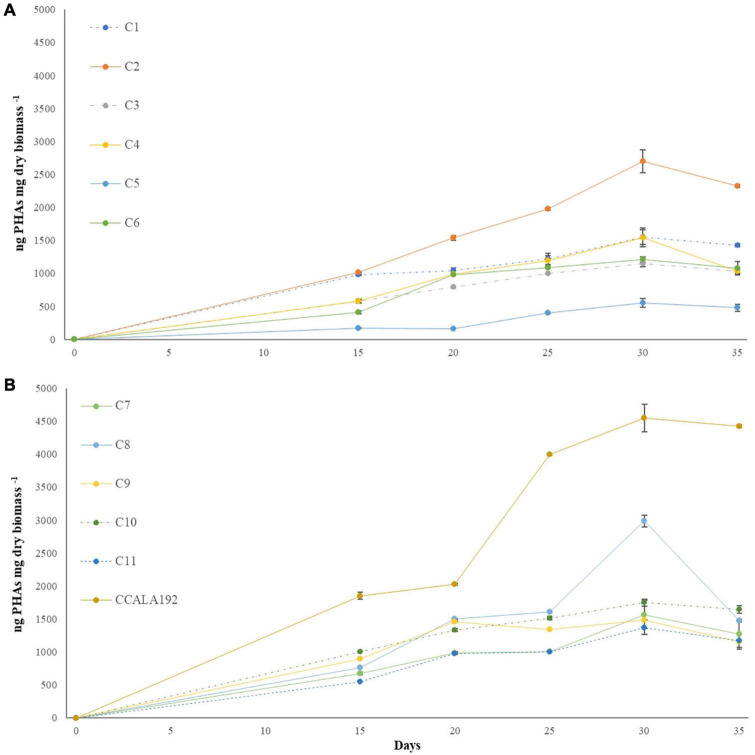
Polyhydroxyalkanoates production curves recorded for “Stiffe” caves’ cyanobacteria and *Synechocystis* cf. *salina*, grown in BG11 modified for starvation. **(A)** C1-C6 curves; **(B)** C7-C11 and CCALA192 curves.

**FIGURE 5:**
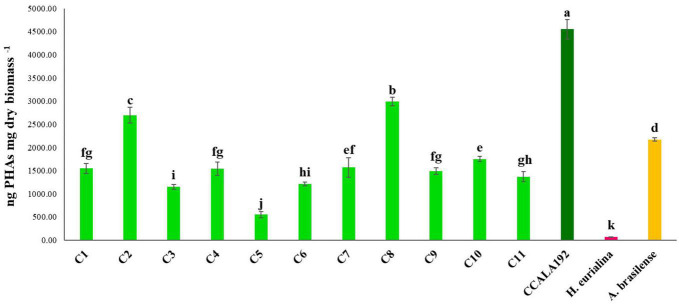
Polyhydroxyalkanoates contents recorded for “Stiffe” caves’ cyanobacteria and *Synechocystis* cf. *salina*, grown in BG11 modified for starvation, and for *A. brasilense* and *H. eurihalina*, cultured on NFCC and MH, respectively. Uppercase letters refer to the comparison among BG11_0_ growths and *A. brasilense* and *H. eurihalina.* Lowercase letters refer to the comparison among BG11 starving growths and *A. brasilense* and *H. eurihalina.* For both conditions, results followed by the same case letters are not significantly different according to Fisher’s LSD *post hoc* test (*p* > 0.05) (LSD value, 192.1).

The eleven isolates were initially checked for their ability to produce PHAs in BG11_0_. Extractions and quantifications of 30 days bioreactions showed that all the tested strains could produce PHAs, with different accumulation rates according to the strain type. To establish the PHA production rates, we cultivated standard strains with renewed PHAs accumulation abilities, namely, *Synechocystis* cf., *A. brasilense*, and *H. eurihalina*. The PHA results obtained for “Stiffe” isolates and *Synechocystis* cf. grown in BG11_0_ and for *A. brasilense* and *H. eurihalina* grown in NFCC and HM, respectively, are reported in Figure 3. The PHA quantities of the cyanobacterial isolates ranged from 266 to 1,152 ng mg dry biomass^–1^. The highest results among the isolates were recorded for strain C3, with amounts significantly lower than *A. brasilense* standard strain (*p* < 0.05). The lowest results among the isolates were recorded for C5 and C6 strains, with amounts significantly lower than *Synechocystis* cf. but still higher than *H. eurihalina* (*p* < 0.05). The other isolates, C1, C2, C4, and C7–C11, produced PHA amounts higher than *Synechocystis* cf., with an average production rate of 617 ng mg dry biomass^–1^. To evaluate the possibility of enhancing PHB production by cyanobacterial strains, isolates and *Synechocystis* cf. bioreactions were optimized by subjecting strains to nitrogen, phosphate, and sulfur starving and acetate addition after 20 days. As shown in Figure 4, all the strains reached a maximum PHA production after 30 days of culturing and after 10 days of adding acetate. After 30 days, some isolates recorded a drastic decrease in the PHAs, while some kept the PHs level almost stationary. For all the strains, on the 30th day, an average fold change of 2.5 was recorded. For each cyanobacterium, PHA amounts recorded in BG11 modified for starvation cultures showed significant increases than BG11_0_ (Student’s *t*-test, *p* < 0.01). The best increases were recorded for CCALA192 (fold change of 9.3), followed by strain C8 (fold change of 4.2). The lowest increases were registered for strain C3 (fold change of 0.2). As depicted in Figure 5, starving and acetate addition allowed CCALA192 to accumulate a quantity of PHAs higher than those recorded for standard strains. Among the isolates, a similar situation was recorded for strain C2 and strain C8, which accumulated PHA amounts higher than those of *A. brasilense* (*p* < 0.05). The other cyanobacterial isolates recorded PHA amounts lower than *A. brasilense* but higher than *H. eurihalina* (*p* < 0.05).

### Polyhydroxyalkanoate characterization by attenuated total reflectance-fourier transform infrared

**FIGURE 6:**
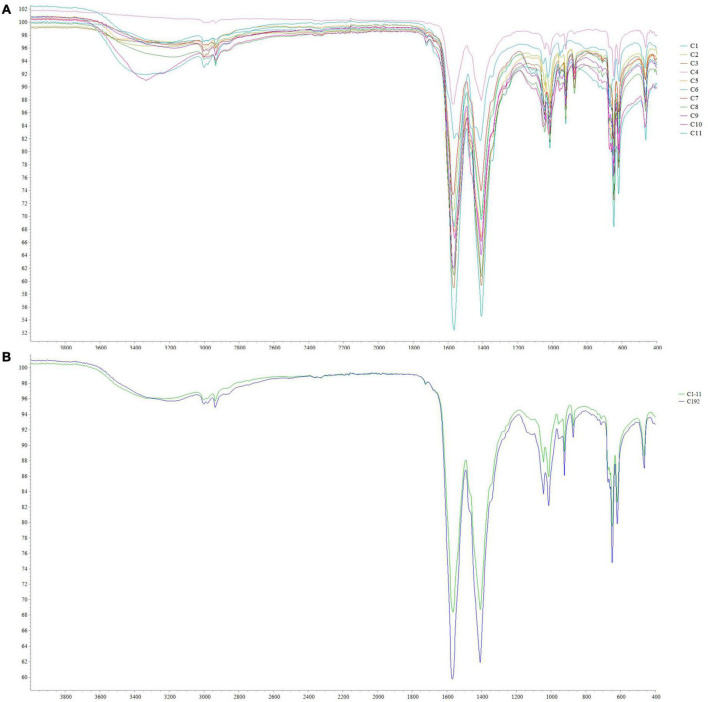
ATR-FTIR spectra obtained from C1–C11 isolates **(A)** and comparison between the average spectrum obtained for C1–C11 isolates with that obtained for *Synechocystis* cf. *salina* (C192) **(B)**.

**FIGURE 7:**
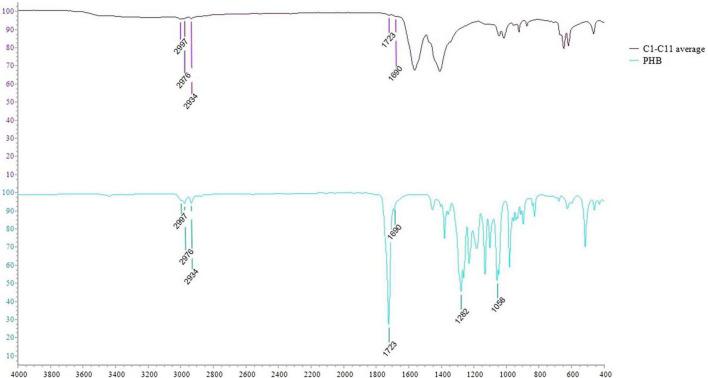
ATR-FTIR spectra comparison between the average spectrum obtained for C1–C11 isolates with that obtained for PHB pure standard.

The PHAs extracted from cyanobacterial isolates were characterized by ATR-FTIR. The spectra obtained for each isolate are shown in Figure 6A. The spectra comparison identified overlapping peaks among isolates and, for this reason, we processed data by creating an average spectrum for C1–C11 isolates. The average spectrum was first compared to that obtained for *Synechocystis* cf. (Figure 6B). The evaluation is performed to underline completely overlapping spectra, suggesting a similar production ability of PHAs and similar compounds within extracts. The C1–C11 average spectrum was also compared to the one acquired for the PHB standard. As presented in Figure 7, the comparison identified within the C1–C11 average spectrum the peaks at 2,997, 2,976, 2,934, 1,723, and 1,690 (transmittance over 5%). These peaks were also observed within the standard polymer spectrum. Due to the presence of other compounds within PHA extracts, the other signals at 1,282 and 1,058 were masked. However, the signals recorded suggested the presence of PHB polymer within the extracts.

### Determination of photosynthetic contents

The biomass obtained from cyanobacterial bioreactions under normal and starving conditions was investigated for the photosynthetic pigments’ contents. Table 2 shows the contents of chlorophyll a of cyanobacteria from “Stiffe” cave and *Synechocystis* cf. *salina* cultivated in BG11_0_ and BG11 modified for starvation. In BG11_0_, chlorophyll a was 2.3–269.6 μg g fresh weight biomass^–1^. Starving led to a significant decrease in the chlorophyll a production, with a range of 0.3–4.2 μg chlorophyll a g fresh weight biomass^–1^. As reported in Table 3, an opposite behavior was recorded for carotenoids, which were low in BG11_0_ (0.06–5.50 μg carotenoids g fresh weight biomass^–1^) and high in BG11 modified for starvation (up to 114.74 μg carotenoids g fresh weight biomass^–1^). In both conditions, the best production rate was recorded for strain C9.

**TABLE 2:** Chlorophyll *a* contents recorded for “Stiffe” caves’ cyanobacteria and *Synechocystis* cf. *salina*, grown in BG11_0_ and BG11 modified for starvation.

	Chlorophyll *a* (μg g fresh weight biomass ^–1^)		
Strain	BG11	BG11 m	*t*-Student *P*-value
C1	24.87 ± 0.93de	4.18 ± 0.28a	*
C2	25.14 ± 1.87de	0.29 ± 0.01gh	**
C3	8.48 ± 0.39fg	0.73 ± 0.02f	*
C4	2.74 ± 0.17g	0.26 ± 0.04gh	**
C5	2.28 ± 0.19g	0.08 ± 0.01hi	***
C6	8.97 ± 0.21fg	1.08 ± 0.11e	*
C7	162.28 ± 12.10b	2.51 ± 0.05c	**
C8	34.96 ± 2.82d	0.69 ± 0.11f	**
C9	269.64 ± 22.37a	1.63 ± 0.09d	**
C10	108.82 ± 6.61c	1.65 ± 0.12d	**
C11	16.41 ± 1.63ef	0.38 ± 0.04g	**
CCALA192	113.79 ± 6.47c	3.25 ± 0.39b	**
LSD	12.22	0.24	

For each column, results followed by the same case letter are not significantly different according to Fisher’s LSD *post hoc* test (*p* > 0.05). For each strain, the *p* levels in the right column refer to statistical differences between chlorophylls of BG11 growth and under starving according to Student’s *t*-test (* *p* < 0.001; **p < 0.01; *** *p* < 0.05).

**TABLE 3:** Carotenoids contents recorded for “Stiffe” caves’ cyanobacteria and *Synechocystis* cf. *salina*, grown in BG11_0_ and BG11 modified for starvation.

	Carotenoids (μg g fresh weight biomass ^–1^)		
	BG11	BG11 m	*t*-Student *P*-value
C1	5.50 ± 0.62a	14.93 ± 0.43fg	*
C2	1.99 ± 0.06cd	16.72 ± 1.22f	**
C3	0.39 ± 0.06gh	4.85 ± 0.17ij	*
C4	0.06 ± 0.00hi	8.87 ± 0.73hi	*
C5	0.55 ± 0.09g	0.98 ± 0.03j	**
C6	1.43 ± 0.04ef	10.54 ± 0.38gh	*
C7	2.09 ± 0.24c	60.35 ± 4.45b	**
C8	0.17 ± 0.00hi	23.20 ± 1.68e	**
C9	1.54 ± 0.13e	114.74 ± 9.56a	**
C10	1.73 ± 0.15de	49.41 ± 2.91c	**
C11	1.13 ± 0.06f	12.65 ± 1.20fgh	**
CCALA192	4.88 ± 0.23b	43.25 ± 2.81d	**
LSD	0.33	5.17	

For each column, results followed by the same case letter are not significantly different according to Fisher’s LSD *post hoc* test (*p* > 0.05). For each strain, the *p* levels in the right column refer to statistical differences between carotenoids of BG11 growth and under starving according to Student’s *t*-test (*p < 0.001; **p < 0.01).

## Discussion

The search for novel biomolecules is based on the selection of microorganisms that express new chemistry due to various extreme environmental conditions (Panou and Gkelis, 2022). Caves are energy-poor ecosystems characterized by high humidity, low natural light, spatial confinement, climatic stability, and low biodiversity (Lamprinou et al., 2012; Culver and Pipan, 2019). For this reason, these ecosystems are very sensitive to anthropogenic pressures (Mammola et al., 2019), which can promote the development of microflora with peculiar characteristics. Some caves are used as tourist attractions or as *in situ* scientific laboratories. The anthropogenic pressure generated by these activities leads to a change in microbial communities, which are extremely sensitive to environmental changes. Among the factors that most induce changes in microbial communities is electric lighting. Introducing light in caves favors the development of biofilms and mats of photosynthetic microorganisms on the surface of walls and speleothems. These communities have been studied to devise strategies and light sources that can limit their formation. However, few studies have investigated the characteristics that microorganisms develop in these extreme environments. This study focused on cyanobacterial lampenflora and its ability to produce polymers and pigments.

The 16S rRNA gene metabarcoding of the lampenflora bacterial and archaeal community confirmed the presence of cyanobacteria within the community. The most abundant genus was *Cyanobium*, a genus already described in Sybil’s Cave (Naples, Italy), colonizing high light intensity sites (Cennamo et al., 2012). This genus was also described in Saint Cave (Licodia Eubea, Catania, Italy) by Di Carlo et al. (2016) who found it colonizing pigmented biofilm sited in several mural areas of Saints Cave (Palermo, Italy) (Di Carlo et al., 2016), and Palla et al. (2012) described its presence in a pigmented biofilm covering the “*Antro delle Sepolture*” surfaces. Metabarcoding also highlighted the presence of SAR324 clade (Marine_group_B). The presence of this marine taxon within the cave ecosystem is quite unusual and highly likely induced by anthropogenic pressure to which the cave has been subjected (Zhelyazkova et al., 2020). The SAR324 clade was described for the first time in the Sargasso Sea (Wright et al., 1997). The distribution of this taxon is relevant in deep waters and oceans (DeLong et al., 2006; Brown and Donachie, 2007; Ghiglione et al., 2012; Dick et al., 2013; Boeuf et al., 2021; Flood et al., 2021) and is a major component of low-oxygen environments, under dysoxic and suboxic conditions (Boeuf et al., 2021). The SAR324 reports underline a high flexible metabolism and a chemoautotrophic regime (Boeuf et al., 2021). Metagenome data support a flexible lifestyle, with carbon monoxide and methane oxidation, methylotrophy, adhesion, and motility genes (Yilmaz et al., 2016). The anthropogenic pressure underlined is also in line with the findings of Manenti et al. (2018) who reported considerable alterations to the stream that runs through the cave, including dam construction and stream bed modifications. During their survey, they also found signs of water contamination in the form of widespread periphyton covering on the stream’s bottom (Manenti et al., 2018). The other representative ASVs belonged to the *Crossiella* genus, a dominant member of the microbial communities of speleothems (Riquelme et al., 2015; Jurado et al., 2020; Miller et al., 2020). The other ASVs belonged to unknown and uncultured taxa mainly associated with Gammaproteobacteria, another common phylum of environments of caves. The Pasteurellaceae family, which mainly constituted the unknown community, is usually described as part of the bat guano bacterial community (Newman et al., 2018).

The isolates tested are good producers of PHAs, especially under starvation. The best PHA producers were strains C2 and C8. The PHA production ability is relevant in the new biopolymers search, which falls among the goals of the 2030 Agenda for Sustainable Development. The non-biodegradable plastics are threatening the environment by accumulating petrochemical derivatives in soil and water (Jambeck et al., 2015; Gomes Gradíssimo et al., 2020). The PHAs have physicochemical characteristics in comparable with petrochemical plastics (Philip et al., 2007; Abed et al., 2009; Geyer et al., 2017), are easily handled with widespread industrial techniques (Wu et al., 2001), and are entirely mineralized into water and carbon dioxide by the action of natural microorganisms (Williams et al., 1999). These characteristics make PHAs a valuable alternative to non-biodegradable plastics, which is of industrial interest for several applications.

The chemical characterization of PHA extracts suggested the presence of PHB within biomass extracts. Bioplastics have been widely studied and applied for their shorter degradation time when exposed to a biologically active environment during the last decades. Among biopolymers, PHB has been investigated for food packaging purposes. Dimensional and mechanical tests showed that PHB could be a good substitute for polypropylene (PP) to produce food packaging. Compared to PP, PHB has a different resistance to dynamic compression, and the deformation value is 50% lower than PP, defining a more rigid and less flexible material. PHB resists higher temperatures than PP while recording lower performances at low-temperature conditions (Bucci et al., 2005). However, the limitations of some studies address the cost and performance of blending PHB with certain polyethers, polyesters, polyvinylacrylates, and polysaccharides in ways that improve mechanical properties without affecting the biodegradability advantage. A wide range of properties emerged from the blending, such as crystallinity, glass transition, and melting temperatures, also posing the hypotheses on the possible “driving force” that such a blend takes (Avella et al., 2000). PHB has also been investigated in the medical field for long-range repair in peripheral nerves and cartilage tissue engineering.

Autologous nerve grafting remains the treatment of choice for peripheral nerve injury repair. However, progress is being made concerning the use of PHB to fill long nerve gaps (up to 4 cm) in a model of peroneal nerve injury (Young et al., 2002). The tests were performed on rabbits, and regeneration was evaluated for 63 days. By day 42, the area of immune-stained regenerating fibers in the PHB group was more significant than in the nerve autograft group, suggesting their adaptability for this type of repair. PHB had no cytotoxicity on mice and the production of a cartilage-like tissue for 24 weeks after implantation was observed (Ye et al., 2009).

Due to their reduced productivity compared to heterotrophic bacteria, PHB production by cyanobacteria is feasible if paired with the production of other metabolites. The presence of valuable by-products within industrial productions is of interest for cost reduction. Our findings underlined that the isolates could also be a good source of chlorophyll a and carotenoids, under normal and starving conditions, respectively. Pigments, especially chlorophylls and carotenoids, are considered key bioactive chemicals (Hosikian et al., 2010). In biotechnology, pigments can be used as a natural dye (Timberlake and Henry, 1986) and in cosmetic and pharmaceutical products (da Silva Ferreira and Sant’Anna, 2017). Chlorophyll a has interesting antioxidant and anti-inflammatory properties (Subramoniam et al., 2012; da Silva Ferreira and Sant’Anna, 2017). Carotenoids are the most diverse group of pigments found in living organisms (Hirschberg and Chamovitz, 1994). It is estimated that around 600 pigments are classified as carotenoids (Guedes et al., 2011). They absorb light during the photosynthesis process and maintain thylakoid membranes and provide a photoprotection as well as the elimination of reactive oxygen species (ROS) (Lawlor, 1995; Schagerl and Müller, 2006; Pagels et al., 2021). Microalgae carotenoids have a great interest in the industry, food, cosmetics, and pharmaceutical applications due to their bioactive properties as an antioxidant, anti-inflammatory, and antitumor (Guedes et al., 2011; Raposo et al., 2015; Cezare-Gomes et al., 2019; Pagels et al., 2020). Several carotenoids are used in agriculture for soil remediation as antioxidants, fertilizers, and biopesticides, to improve soil quality and crop protection. At present, they are used to increase provitamin A availability contributing to biofortified crop development (Sakamoto et al., 2017; Gonçalves, 2021).

## Conclusion

“Stiffe” caves’ greenish mats and biofilms 16S rRNA gene metabarcoding underlined anthropogenic pressure-driven bacterial and archaeal communities’ alterations. However, the lightning sources promoted the proliferation of culturable cyanobacterial strains with good PHB accumulation abilities (266 to 1,152 ng mg dry biomass^–1^). The optimization of bioprocesses by nutrient starving improved PHA accumulation, with an average fold change of 2.5. The FTIR analyses suggested the PHB presence within PHAs extracted by cyanobacteria isolates. The comparison of cyanobacterial bioprocesses with those of *A. brasilense* and *H. eurihalina* showed that even if the optimal bioprocess last 30 days, cyanobacteria can produce PHA amounts employing a lower amount of nutrients and producing bioproducts with interesting biological properties. The isolation of these cyanobacteria that produces these large quantities of PHB could be linked to the critical environmental conditions of the lack of light for an extended period of closure linked to the COVID-19. PHB accumulation in cyanobacteria allows coping with unfavorable environmental conditions. Reasonably, during the switch-off, the PHB-producing cyanobacteria with high resilience and adaptability were selected by adverse environmental conditions.

Further studies on cyanobacterial bioprocesses and characterizations of PHB should be carried out to understand the biotechnological potential at the industrial level. The optimal bioreaction parameters should be studied to maximize the PHB production and pigments (e.g., temperature, effects of different lights, and light intensity). The molecular identification of the isolates and the study of the PHA production pathway gene expression will also clarify the biotechnological potential of the selected isolates. However, the findings broadened the knowledge of the microbial communities of mats and biofilms on the lightened walls of the “Stiffe” caves. The results showed that these formations could be possible sources of biomolecules and biopolymers, underlying the importance of exploring their potentialities before taking device strategies.

## Data availability statement

The datasets generated and analyzed during this study are available from the corresponding author on reasonable request. The nucleotide sequences of the partial 16S rRNA gene segments determined in this study have been deposited in the NCBI database repository, BioProject: PRJNA833652 (http://www.ncbi.nlm.nih.gov/bioproject/833652).

## Author contributions

IV, RD, and MP performed the samplings. RD and AM carried out the experiments and analyses. MP and DS coordinated the research activities. MP, RD, and AM handled the data and performed the results interpretation. RD, IV, and DS wrote the original draft of the manuscript. AD’A, MP, and MD performed the manuscript revision. MP handled manuscript processing and correspondence. MP, DS, AD’A, and MD conceptualized the experiments. AD’A, DS, and MD coordinated the project. All authors contributed to the article and approved the submitted version.

## Footnotes

1 https://www.arb-silva.de/ (accessed on October 2021).
