# Supplementary material for: Polyhydroxybutyrate-producing cyanobacteria from lampenflora: The case study of the “Stiffe” caves in Italy
Source: Front Microbiol. 2022 Jul 28;13:933398. doi: 10.3389/fmicb.2022.933398 (PMC9366245; doi:10.3389/fmicb.2022.933398)
Supplement: Supplementary file 1 [file Data_Sheet_1.docx]

Supplementary material

Polyhydroxybutyrate-producing cyanobacteria from lampenﬂora: The case study of the “Stiffe” caves in Italy

Rihab Djebaili^1^, Amedeo Mignini^1^, Ilaria Vaccarelli^1^, Marika Pellegrini^1,*^, Daniela M. Spera^2^, Maddalena Del Gallo^1^, Anna Maria D’Alessandro^1,*^

^1^Department of Life, Health and Environmental Sciences, University of L'Aquila, L'Aquila, Italy

^2^Quality Engineering S.r.l., Pescara, Italy

*** Correspondence:**Marika Pellegrini
marika.pellegrini@univaq.it

Anna Maria D’Alessandro

annamaria.dalessandro@univaq.it

Figure S1 – Optical microscope morphologies identified for the isolates and associated to *Synechocystis* and *Synechococcus,* genera.


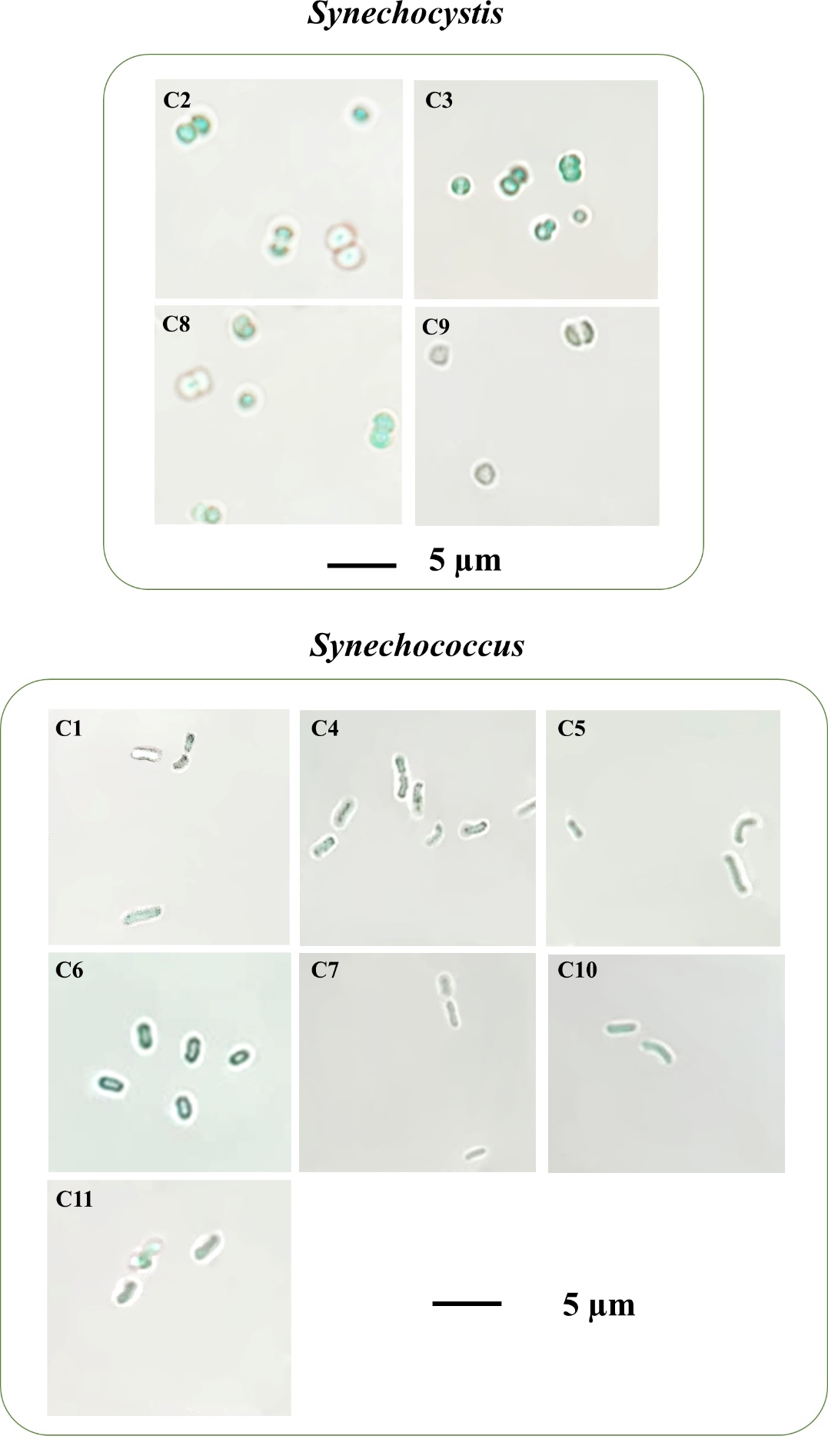


Table S1 - ASVs abundances and taxonomic assignments at phylum level.

| **Domain** | **Phylum** | **Abundances*** |
| --- | --- | --- |
| Bacteria | Acidobacteriota | 9.7 |
|  | Actinobacteriota | 11.3 |
|  | Bacteroidota | 7.7 |
|  | Campylobacterota | 0.6 |
|  | Chloroflexi | 4.4 |
|  | Cyanobacteria | 0.9 |
|  | Dadabacteria | 0.9 |
|  | Desulfobacterota | 0.7 |
|  | Elusimicrobiota | 1.0 |
|  | Firmicutes | 7.1 |
|  | Fusobacteriota | 1.7 |
|  | Gemmatimonadota | 1.3 |
|  | Latescibacterota | 0.8 |
|  | Methylomirabilota | 1.1 |
|  | Myxococcota | 2.7 |
| Archaea | Nanoarchaeota | 0.7 |
| Bacteria | NB1-j | 1.1 |
|  | Nitrospirota | 3.4 |
|  | Patescibacteria | 2.4 |
|  | Planctomycetota | 3.4 |
|  | Proteobacteria | 32.6 |
|  | SAR324 | 0.7 |
|  | Verrucomicrobiota | 3.9 |

In the Table: * Abundances > 0.5%; SAR324, SAR324_clade (Marine_group_B).

Table S2 - ASVs abundances and taxonomic assignments at genus level.

| **Domain** | **Phylum** | **Class** | **Order** | **Family** | **Genus** | **Abundances*** |
| --- | --- | --- | --- | --- | --- | --- |
| Bacteria | Acidobacteriota | Blastocatellia | 11-24 | 11-24 | *11-24* | 2.1 |
| Bacteria | Actinobacteriota | Thermoleophilia | Solirubrobacterales | 67-14 | *67-14* | 1.4 |
| Bacteria | Chloroflexi | Anaerolineae | SBR1031 | A4b | *A4b* | 1.0 |
| Bacteria | Proteobacteria | Gammaproteobacteria | Pseudomonadales | Moraxellaceae | *Acinetobacter* | 1.0 |
| Bacteria | Myxococcota | bacteriap25 | bacteriap25 | bacteriap25 | *bacteriap25* | 1.3 |
| Bacteria | Verrucomicrobiota | Omnitrophia | Omnitrophales | Omnitrophaceae | *Candidatus_Omnitrophus* | 1.4 |
| Bacteria | Patescibacteria | Microgenomatia | Candidatus_Woesebacteria | Candidatus_Woesebacteria | *Candidatus_Woesebacteria* | 0.9 |
| Bacteria | Actinobacteriota | Actinobacteria | Pseudonocardiales | Pseudonocardiaceae | *Crossiella* | 9.9 |
| Bacteria |  |  | Propionibacteriales | Propionibacteriaceae | *Cutibacterium* | 0.9 |
| Bacteria | Cyanobacteria | Cyanobacteriia | Synechococcales | Cyanobiaceae | *Cyanobium_PCC-6307* | 0.8 |
| Bacteria | Dadabacteria | Dadabacteriia | Dadabacteriales | Dadabacteriales | *Dadabacteriales* | 1.3 |
| Bacteria | Fusobacteriota | Fusobacteriia | Fusobacteriales | Fusobacteriaceae | *Fusobacterium* | 1.1 |
| Bacteria | Proteobacteria | Gammaproteobacteria | Methylococcales | Methylomonadaceae | *IheB2-23* | 1.3 |
| Bacteria |  |  | Burkholderiales | Nitrosomonadaceae | *IS-44* | 1.1 |
| Bacteria | Latescibacterota | Latescibacterota | Latescibacterota | Latescibacterota | *Latescibacterota* | 1.1 |
| Bacteria | Nitrospirota | Leptospirillia | Leptospirillales | Leptospirillaceae | *Leptospirillum* | 1.6 |
| Bacteria | Fusobacteriota | Fusobacteriia | Fusobacteriales | Leptotrichiaceae | *Leptotrichia* | 1.3 |
| Bacteria | Proteobacteria | Gammaproteobacteria | Burkholderiales | Nitrosomonadaceae | *MND1* | 6.5 |
| Bacteria | NB1-j | NB1-j | NB1-j | NB1-j | *NB1-j* | 1.6 |
| Bacteria | Proteobacteria | Gammaproteobacteria | Burkholderiales | Neisseriaceae | *Neisseria* | 1.2 |
| Bacteria | Nitrospirota | Nitrospiria | Nitrospirales | Nitrospiraceae | *Nitrospira* | 3.5 |
| Bacteria | Planctomycetota | Pla4_lineage | Pla4_lineage | Pla4_lineage | *Pla4_lineage* | 1.0 |
| Bacteria | Bacteroidota | Bacteroidia | Bacteroidales | Porphyromonadaceae | *Porphyromonas* | 1.0 |
| Bacteria |  |  |  | Prevotellaceae | *Prevotella* | 1.6 |
| Bacteria |  |  |  |  | *Prevotella_7* | 2.0 |
| Bacteria | Proteobacteria | Gammaproteobacteria | Pseudomonadales | Pseudomonadaceae | *Pseudomonas* | 1.2 |
| Bacteria | Methylomirabilota | Methylomirabilia | Rokubacteriales | Rokubacteriales | *Rokubacteriales* | 1.5 |
| Bacteria | SAR324 | SAR324 | SAR324 | SAR324 | SAR324 | 1.1 |
| Bacteria | Firmicutes | Bacilli | Staphylococcales | Staphylococcaceae | *Staphylococcus* | 1.3 |
| Bacteria | Proteobacteria | Gammaproteobacteria | Steroidobacterales | Steroidobacteraceae | *Steroidobacter* | 1.1 |
| Bacteria | Firmicutes | Bacilli | Lactobacillales | Streptococcaceae | *Streptococcus* | 1.6 |
| Bacteria | Acidobacteriota | Vicinamibacteria | Subgroup_17 | Subgroup_17 | *Subgroup_17* | 1.0 |
| Bacteria |  | Subgroup_22 | Subgroup_22 | Subgroup_22 | *Subgroup_22* | 0.8 |
| Bacteria | Proteobacteria | Gammaproteobacteria | Burkholderiales | TRA3-20 | *TRA3-20* | 1.8 |
| Bacteria | Chloroflexi | Anaerolineae | Anaerolineales | Anaerolineaceae | *UTCFX1* | 0.8 |
| Bacteria | Firmicutes | Negativicutes | Veillonellales-Selenomonadales | Veillonellaceae | *Veillonella* | 1.1 |
| Bacteria | Acidobacteriota | Vicinamibacteria | Vicinamibacterales | Vicinamibacteraceae | *Vicinamibacteraceae* | 5.9 |
| Bacteria | Proteobacteria | Gammaproteobacteria | Nitrosococcales | Nitrosococcaceae | *wb1-P19* | 1.8 |
| Bacteria |  |  | uncultured | uncultured | uncultured | 22.4 |
| Bacteria |  |  | Enterobacterales | Pasteurellaceae | unknown | 8.6 |

In the Table: * Abundances > 0.5%; SAR324, SAR324_clade (Marine_group_B).
